# Gesetzliches Hautkrebsscreening in Deutschland: Motivation und Motive zur Teilnahme versus Nichtteilnahme

**DOI:** 10.1007/s00105-021-04842-0

**Published:** 2021-07-05

**Authors:** G. Girbig, M. Augustin, M. Krensel, V. Andrees

**Affiliations:** Institut für Versorgungsforschung in der Dermatologie und bei Pflegeberufen (IVDP), Universitätsklinikum Hamburg-Eppendorf (UKE), Martinistr. 52, 20246 Hamburg, Deutschland

**Keywords:** Vorsorgeuntersuchung, Hautkrebsprävention, Inanspruchnahme, Teilnehmerquote, Früherkennungsuntersuchung, Preventive examination, Skin cancer prevention, Health care utilization, Participation rate, Mass screening

## Abstract

**Hintergrund:**

Im Jahr 2008 wurde in Deutschland das gesetzliche Hautkrebsscreening (gHKS) für gesetzlich Versicherte ab 35 Jahren eingeführt. Primäres Ziel ist es, maligne Veränderungen der Haut frühzeitig zu diagnostizieren, die Mortalität zu reduzieren sowie die Morbidität und Lebensqualität zu verbessern. Obwohl das gHKS vor mehr als einer Dekade eingeführt wurde, wird dieses nur von einem Teil wahrgenommen.

**Ziel der Arbeit:**

Welche Gründe bestehen für die geringe Teilnahmequote am gHKS in der berechtigten Normalbevölkerung?

**Methodik:**

In computergestützten Telefoninterviews mit einer repräsentativen Bevölkerungsstichprobe (*N* = 1015) gesetzlich Versicherter ab 18 Jahren wurden im Januar 2019 die Einstellung zum Thema Hautkrebs, die Rate der Inanspruchnahme des gHKS sowie die Gründe für die Nichtteilnahme erhoben. Ein Teil der Daten wurde mit vorausgehenden Erhebungen aus den Jahren 2011, 2013 und 2015 verglichen.

**Ergebnisse:**

Unter den 1015 Teilnehmern wurde das Thema Hautkrebs von 40 % als besorgniserregend eingestuft. Zum gHKS waren 75,4 % der Befragten berechtigt. Von diesen hatten 52,6 % bisher noch nie am gHKS teilgenommen. Die Inanspruchnahme nahm im Alter jedoch zu. Ferner war die Nichtteilnehmerquote bei gehobenem Schulabschluss mit 45 % deutlich niedriger als bei niedrigem Schulabschluss mit 58 %. Der Anspruch auf das gHKS war 35 % der Nichtteilnehmer nicht bekannt. Als Grund für die Nichtteilnahme nannten 20 % Zeitmangel, 58 % sahen keine Notwendigkeit, weil sie sich gesund fühlen. Generell hielten aber 91 % aller 1015 Befragten Früherkennungsuntersuchungen für sinnvoll und hatten zu 66 % bereits an anderen Vorsorgeuntersuchungen teilgenommen.

**Diskussion:**

Die mit etwa 50 % geringe Teilnehmerquote am gHKS sowie die abnehmende Besorgnis in der Bevölkerung um das Thema Hautkrebs legen nahe, dass eine weitere, auch risikogruppenorientierte Aufklärung der Bevölkerung über die Relevanz des Themas Hautkrebs notwendig ist.

## Abstract (en)

**Background:**

In 2008, statutory skin cancer screening (sSCS) was introduced in Germany for people aged ≥ 35 years with statutory health insurance (SHI). The aim is to diagnose malignant skin lesions at an early stage, reduce mortality and improve morbidity and quality of life. Although sSCS was introduced more than a decade ago, only a part of the public is aware of it.

**Objectives:**

What are the reasons for the low participation rate in sSCS in the eligible population?

**Materials and methods:**

In computer-assisted phone interviews with a representative population sample of *N* = 1015 persons aged ≥ 18 years with SHI attitude towards skin cancer, rate of use of sSCS and reasons for nonparticipation were surveyed in January 2019. Parts of the data were compared with previous surveys from 2011, 2013 and 2015.

**Results:**

Among the 1015 participants 40% were concerned about skin cancer (2011: 45%; 2013: 51%; 2015: 46%). Of the respondents 75.4% were authorised to use sSCS. Of these 52.6% had never participated in sSCS before. However, participation increased with age. Further, the nonparticipation rate for those with a high school-leaving certificate (45%) was significantly lower than for those with a lower schooling level (58%). The option for sSCS was unknown to 35% of nonparticipants. As a reason for nonparticipation 20% mentioned a lack of time, while 58% saw no need for it because they felt healthy. In general, 91% of all respondents considered early preventive examinations to be useful and 66% had already taken part in other preventive examinations.

**Conclusions:**

The low participation rate of about 50% in sSCS and the decreasing concern about skin cancer suggest that further education of the general population about the relevance of skin cancer with a focus on risk groups is necessary.

Im Jahr 2008 wurde in Deutschland erstmals das gesetzliche Hautkrebsscreening (gHKS) für gesetzlich Versicherte ab 35 Jahren eingeführt. Ziel ist es, maligne Veränderungen der Haut bzw. deren Vorstufen frühzeitig zu detektieren und durch entsprechende therapeutische Maßnahmen die Mortalität zu senken. Seit Beginn des gHKS ist bereits ein Anstieg der Inzidenz des Hautkrebses zu verzeichnen, jedoch v. a. die der frühen und damit prognostisch besseren Stadien.

Obwohl das gHKS seit dreizehn Jahren von der gesetzlichen Krankenkasse erstattet wird, wird diese Möglichkeit nur von etwa jedem Zweiten wahrgenommen. In dieser Studie wurden die Inanspruchnahme und die Gründe für die Nichtteilnahme evaluiert.

## Hintergrund

Hautkrebs zählt sowohl in Deutschland als auch weltweit zu den häufigsten Krebsarten [1, 2]. Das Tumorstadium zum Zeitpunkt der Diagnosestellung nimmt erheblichen Einfluss auf die Überlebensrate, insbesondere beim malignen Melanom (MM). Primäres Ziel des Hautkrebsscreenings (HKS) ist es, maligne Veränderungen der Haut bzw. deren Vorstufen frühzeitig zu detektieren und durch entsprechende therapeutische Maßnahmen die Mortalität zu senken [3, 4]. Nachdem sich in der Pilotstudie SCREEN zum HKS 2003/2004 in Schleswig-Holstein Hinweise auf eine Reduktion der melanombedingten Mortalität gezeigt hatten, wurde im Sommer 2008 in Deutschland erstmals flächendeckend das HKS für gesetzlich Versicherte ab 35 Jahren eingeführt [3–5]. Hierfür ist eine visuelle Inspektion des gesamten Integuments und der einsehbaren Schleimhäute alle zwei Jahre durch einen Dermatologen oder Allgemeinmediziner nach entsprechender Zusatzausbildung vorgesehen. Die additive Untersuchung mittels Dermatoskopie ist seit April 2020 in den einheitlichen Bewertungsmaßstab-Leistungskatalog (EBM) der gesetzlichen Krankenkassen (GKV) mit aufgenommen. Bisher galt diese als individuelle Gesundheitsleistung (IGeL) [6].

Seit Einführung des Screenings ist bereits ein Anstieg der Inzidenz des Hautkrebses zu verzeichnen, v. a. der frühen und damit prognostisch günstigeren Stadien [7]. Ferner zeigt sich zwischen 2013 und 2017 ein Rückgang der Mortalität am MM unter Altersstandardisierung [8, 9].

Das gHKS ist eine sinnvolle, für den Patienten wenig aufwendige Maßnahme, die bei einem großen Teil der Bevölkerung auf Zustimmung trifft [10]. Dennoch ist die Teilnahmequote verhalten, nur etwa jeder zweite Berechtigte nahm die Leistung bisher in Anspruch [11]. Nach dreizehn Jahren gHKS wollen wir die Gründe für die geringe Inanspruchnahme beleuchten.

Die Studie untersucht die allgemeine Wahrnehmung des Themas Hautkrebs in der Bevölkerung, die Population der Nichtteilnehmer am gHKS und deren Gründe für die Nichtteilnahme trotz Berechtigung.

## Methodik

In computergestützten Telefoninterviews wurde eine repräsentative Bevölkerungsstichprobe von deutschsprachigen gesetzlich Versicherten ab 18 Jahren genommen und zum gHKS befragt. Diese Querschnitterhebung erfolgte im Zeitraum vom 22.01.2019 bis 01.02.2019 in der gesamten Bundesrepublik Deutschland durch entsprechend ausgebildete Mitarbeiter der Forsa Gesellschaft für Sozialforschung und statistische Analysen mbH.

Inhaltlich wurde nach der Meinung der Allgemeinbevölkerung zum gHKS und anderen Vorsorgeuntersuchungen bzw. Früherkennungsuntersuchungen gefragt sowie danach, inwieweit die Befragten über ihren Zugang und ihre Berechtigung informiert waren. Bei den Nichtteilnehmern des gHKS, die ab 35 Jahren jedoch dazu berechtigt sind, wurde nach den Beweggründen für die Nichtteilnahme gefragt. Ein Teil der Daten wurde mit vorausgehenden Erhebungen aus den Jahren 2011, 2013 und 2015 verglichen.

Die statistische Auswertung erfolgte mithilfe der Statistiksoftware IBM SPSS Statistics Version 23 (Armonk, NY, USA). Um ein repräsentatives Meinungsbild der Gesamtbevölkerung zu erhalten, wurde ein Gewichtungsfaktor für Alter, Geschlecht, Bildungsstatus und Region mit einbezogen. Die deskriptive Datenauswertung erfolgte zum Gesamtkollektiv beim allgemeinen Meinungsbild zu Vorsorgeuntersuchungen. Ferner wurden die Daten zur Gruppe der gHKS-Berechtigten ab 35 Jahren mit der Subgruppe der Nichtteilnehmer zu den Gründen der Nichtteilnahme deskriptiv ausgewertet. Hierbei wurde unter anderem auf die soziodemografischen Variablen Geschlecht (männlich, weiblich; divers zum Erhebungszeitpunkt noch nicht gefordert), Alter (35 bis 64 Jahre, > 64 Jahre), Bildungsniveau (niedrig, mittel, hoch), Region (Westdeutschland: alte Bundesländer inklusive Berlin, Ostdeutschland: neue Bundesländer exklusive Berlin) und Regionstyp (städtische Region, Region mit Verstädterungsansätzen, ländliche Region) eingegangen. „Weiß nicht“ sowie „keine Angabe“ als Antworten wurden als fehlende Antworten gewertet und nicht in die Berechnung einbezogen. Mit dem Chi-Quadrat(χ^2^)-Test wurden Gruppenunterschiede auf ihre statistische Signifikanz mit einem Signifikanzniveau von α = 0,05 überprüft.

## Ergebnisse

### Wahrnehmung in der Allgemeinbevölkerung

Insgesamt 1015 gesetzlich Versicherte ab 18 Jahren nahmen an der Studie teil, davon 531 (52,3 %) Frauen (Tab. 1). Zur Teilnahme am gHKS waren mit mindestens 35 Jahren 765 der Teilnehmer (75,4 %) berechtigt.

**Table 1:** 

Befragte (*N* = 1015)	
*Männer*	484 (47,7 %)
*Frauen*	531 (52,3 %)
*Alter (n* *=* *1015)*	
< 35 Jahre	251 (24,7 %)
≥ 35 Jahre	765 (75,4 %)
35 bis 64 Jahre	490 (48,3 %)
> 64 Jahre	274 (27,0 %)
*Bildungsniveau (n* *=* *985)*	
Niedrig	391 (39,7 %)
Mittel	311 (31,5 %)
Hoch	283 (28,8 %)
*Region (n* *=* *1015)*	
Ostdeutschland	169 (16,6 %)
Westdeutschland	846 (83,4 %)
*Regionstyp (n* *=* *987)*	
Städtische Region	450 (45,6 %)
Regionen mit Verstädterungsansätzen	331 (33,5 %)
Ländliche Region	206 (20,8 %)

*n* Anzahl der Personen

**Figure 1:**
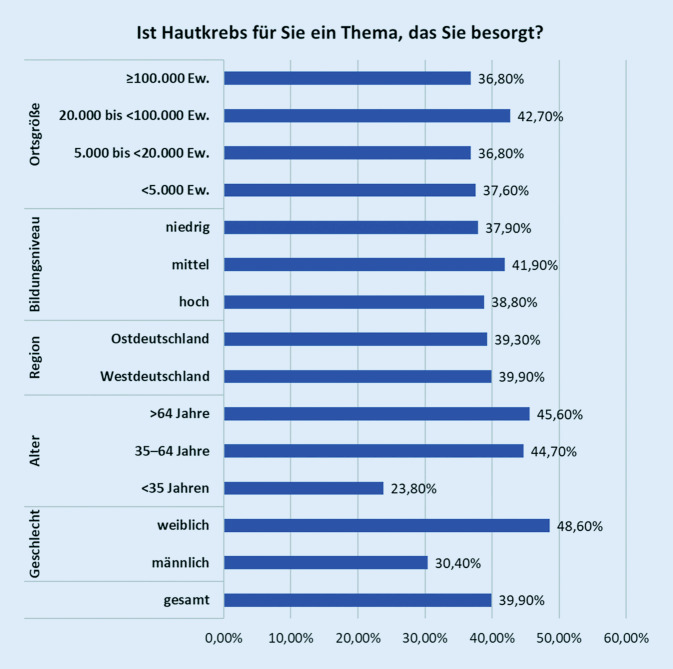

Ein Großteil (60 %) der Befragten empfand Hautkrebs nicht als ein besorgniserregendes Thema. Dies traf insbesondere bei Personen unter 35 Jahren und Männern zu. Im Vergleich zu den Vorjahren war der Teil der Befragten, der Hautkrebs als besorgniserregend einstuft, gesunken: 2011 war das Thema Hautkrebs für 45 %, 2013 für 51 % und 2015 für 46 % besorgniserregend. Bei der aktuellen Umfrage waren es 40 % (Abb. 1).

**Figure 2:**
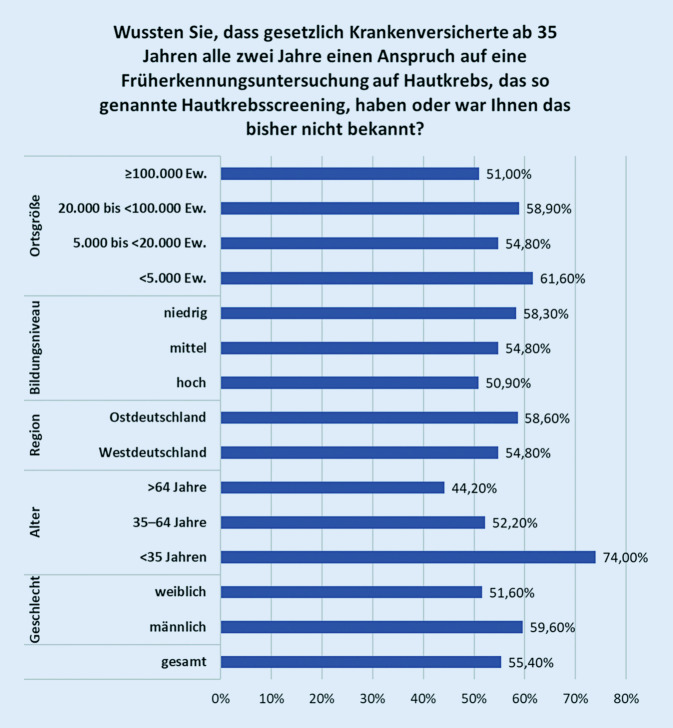

Dass das gHKS alle zwei Jahre in Anspruch genommen werden kann, war 55 % der Befragten nicht bekannt (Abb. 2). Es ist zu beobachten, dass die Kenntnis über die Berechtigung zum gHKS bei Befragten aus ländlichen Regionen weniger verbreitet ist. Ferner stieg die Kenntnis hierüber sprunghaft von 26 % bei den 18- bis 34-Jährigen auf 47,8 % in der Altersgruppe von 35 bis 64 Jahren an, also der Altersgruppe mit Berechtigung. Bei den über 65-Jährigen waren 55,8 % über das gHKS-Angebot informiert. Insgesamt hatten bereits 66 % der Befragten an anderweitigen Vorsorgeuntersuchungen teilgenommen. In der Gruppe der 55- bis 75-Jährigen hatten bereits 80 % andere Vorsorgeuntersuchungen beim Hausarzt wahrgenommen, davon etwas mehr Frauen als Männer.

Generell wurden Vorsorgeuntersuchungen von 91 % der Befragten als sinnvolle Maßnahme erachtet. Dass Vorsorgeuntersuchungen nur genutzt werden sollten, wenn man bereits eine Erkrankung befürchtet, wurde von 38,2 % der unter 35-Jährigen und wieder von 39,3 % der über 65-Jährigen so gesehen. Die Altersklassen dazwischen stimmten zu 75,1 % der Aussage nicht zu.

Von den 765 Befragten mit gHKS-Anspruch hatten bisher 47 % zumindest einmalig daran teilgenommen. Die Teilnahmequote war bei Männern, in Gemeinden unter 5000 Einwohner und in Ostdeutschland mit jeweils etwa 40 % signifikant geringer (Tab. 2).

**Table 2:** 

	Nichtteilnehmer (berechtigt) (*n* = 402)	gHKS-Teilnehmer (jemals) (*n* = 359)	*p*-Wert
*Geschlecht (n* *=* *402)*			0,000*
Männer	213 (53,1 %)	137 (38,2 %)	–
Frauen	189 (46,9 %)	222 (61,8 %)	–
*Alter (n* *=* *402)*			0,076
< 35 Jahre	0 (da nicht berechtigt)	0	–
35 bis 64 Jahre	269 (66,9 %)	218 (60,8 %)	–
> 64 Jahre	133 (33,1 %)	141 (39,2 %)	–
*Bildungsniveau (n* *=* *392)*			0,030*
Niedrig	176 (44,9 %)	126 (35,8 %)	–
Mittel	130 (33,2 %)	121 (34,4 %)	–
Hoch	86 (21,4 %)	105 (29,7 %)	–
*Region (n* *=* *402)*			0,040*
Ostdeutschland	81 (20,2 %)	52 (14,4 %)	–
Westdeutschland	321 (79,8 %)	307 (85,6 %)	–
*Regionstyp (n* *=* *390)*			0,024*
Städtische Region	167 (42,8 %)	163 (45,7 %)	–
Regionen mit Verstädterungsansätzen	121 (31,0 %)	123 (34,4 %)	–
Ländliche Region	102 (26,2 %)	71 (19,8 %)	–
*Arzt (n* *=* *349)*			–
Hausarzt	–	84 (23,9 %)	–
Hautarzt	–	266 (76,1 %)	–

*n* Anzahl der Personen

*p-Wert statistisch signifikant

### Nichtteilnehmerbefragung

**Figure 3:**
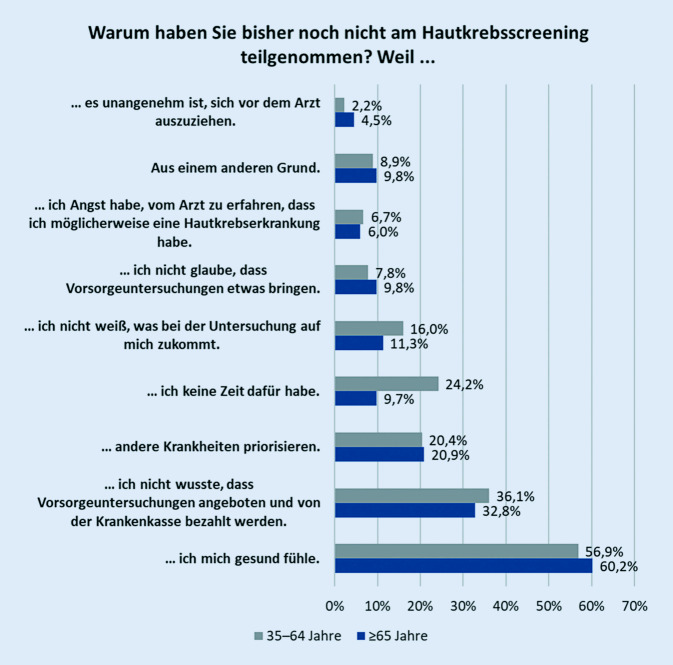

Von den 765 Berechtigten hatten 402 (52,6 %) bisher nicht am gHKS teilgenommen. Als Grund für eine Nichtteilnahme wurde mit 58 % am häufigsten genannt, sich gesund zu fühlen und deshalb noch nicht am Screening teilgenommen zu haben. Diese Antwort erfolgte nahezu alters- und geschlechtsunabhängig, aber eher von Personen mit niedrigem Bildungsabschluss (Abb. 3, 4 und 5). Von den Nichtteilnehmern wussten 34,9 % nicht, dass sie ein Hautkrebsscreening überhaupt in Anspruch nehmen können, das entsprach 37,1 % der Männer und 32,3 % der Frauen. Im berufstätigen Alter (35 bis 64 Jahre) gaben 24,2 % Zeitmangel als Begründung für eine Nichtteilnahme an, ab 65 Jahren taten dies noch 9,7 %. Männer führten das Argument des Zeitmangels deutlich häufiger als Frauen an (22 % und 15,9 %). Ebenso gaben Nichtteilnehmer mit hohem und niedrigem Bildungsstand sowie Ostdeutsche häufiger als Befragte vom mittleren Bildungsstand und Westdeutsche den Zeitmangel als Begründung an (Abb. 6). Dass andere Erkrankungen Priorität hätten, gaben 20,6 % der Befragten an. Der Ablauf bzw. nicht zu wissen, wie genau ein HKS durchgeführt wird, schien für 14,6 % abschreckend zu sein, 2,2 % wollten sich nicht vor dem Arzt ausziehen. Als sinnlos bezeichneten 8,6 % der Nichtteilnehmer das gHKS. Weitere 9,2 % sahen noch andere Gründe für die Nichtteilnahme als relevant an.

**Figure 4:**
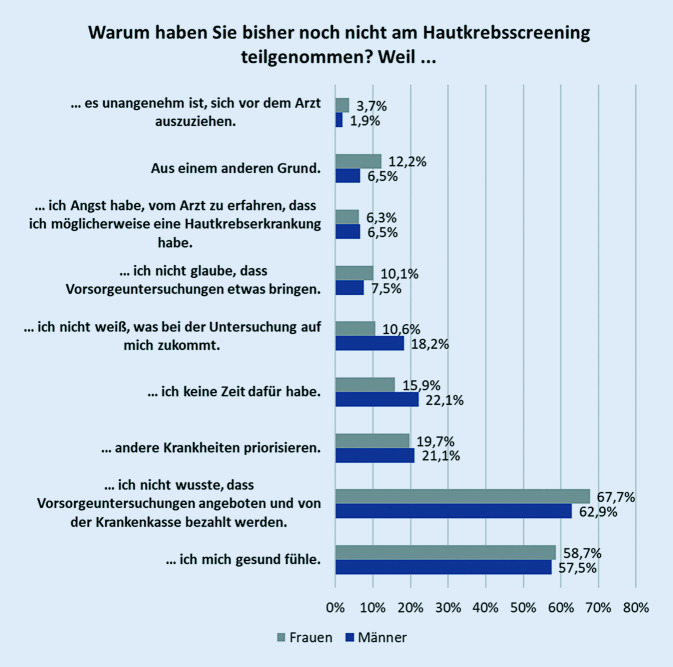

**Figure 5:**
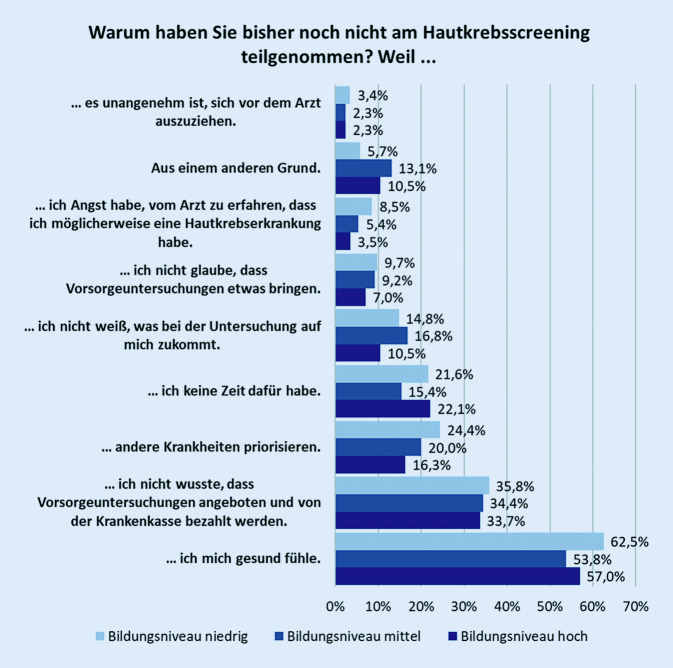

**Figure 6:**
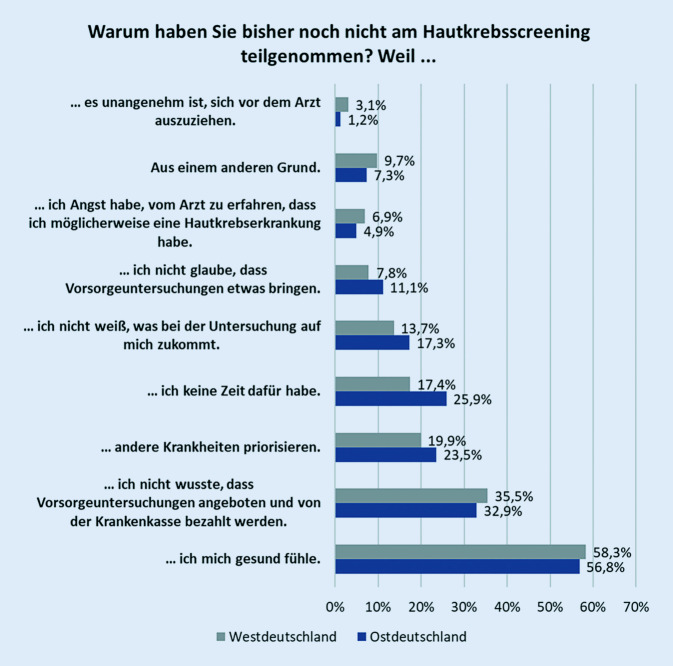

## Diskussion

Ziel der vorliegenden Studie war es, Unterschiede zwischen den Teilnehmenden und den Nichtteilnehmenden am gHKS zu analysieren und die Nichtteilnehmer mit soziodemografischen und Einstellungsvariablen zu charakterisieren. Diese Fragestellung ist insofern von versorgungsrelevanter Bedeutung, als dass bisher nur ein kleinerer Teil der Berechtigten ab 35 Jahren das gHKS in Anspruch genommen hat. Zum einen haben eine weite Anfahrt und lange Wartezeiten beim Facharzt einen organisatorischen und regionalen Einfluss auf die Teilnahme am gHKS, mit dem sich Andrees et al. ausführlich befasst haben [12]. Zum anderen geht die niedrige Teilnehmerzahl mit der beobachteten abnehmenden Besorgnis in der Bevölkerung bezüglich des Themas Hautkrebs einher, obwohl die Mortalität insgesamt steigt [8]. Bei einer Altersstandardisierung zeigte sich jedoch ein Rückgang der Mortalität am MM zwischen 2013 und 2017, der sich evtl. auf die Einführung des gHKS zurückführen lässt [9]. Generell wird der Einfluss aller Krebsscreenings auf die Mortalität als gering, aber als klinisch und gesundheitsökonomisch relevant erachtet [13]. Da viele Befragte angaben, dass sie keine Kenntnisse über eine gHKS-Berechtigung hatten oder dass sie aufgrund von anderen priorisierten Erkrankungen bzw. Zeitmangel nicht teilgenommen hatten, scheinen ein Informationsdefizit und ein damit einhergehendes mangelhaftes Risikobewusstsein über die Gefahr von Hautkrebs in der Bevölkerung zu bestehen. Ähnliche Ergebnisse hinsichtlich eines geringen Risikobewusstseins zum Thema Hautkrebs fanden sich auch in anderen Studien, darunter einer europaweiten Befragung von MacKie und einer Erhebung an Bergführern in Deutschland von Zink et al. [14, 15]. In einer weiteren Studie zeigten sogar bereits an Hautkrebs Erkrankte ein nur geringes Interesse an hautkrebsbezogenen Apps [16].

Weitere Aufklärung der Bevölkerung bezüglich Relevanz des Themas Hautkrebs mit seinem potenziell letalen oder stark beeinträchtigenden Ausgang ist notwendig. Mit einer solchen für den Patienten wenig aufwendigen Vorsorgeuntersuchung können maligne Veränderungen der Haut bzw. deren Vorstufen frühzeitig diagnostiziert und der Verlauf kann günstig beeinflusst werden [3, 5]. Ein besseres Verständnis des Präventionsansatzes ist in der Aufklärungsarbeit dringend zu vermitteln, um gerade diejenigen anzusprechen, die angaben, sich gesund zu fühlen und deshalb nicht am gHKS teilgenommen zu haben. Ferner darf die eigenverantwortliche Selbstuntersuchung als Präventionsmaßnahme nicht unterschätzt werden und muss ebenso in die Aufklärungsarbeit mit einhergehendem Verweis an das gHKS integriert werden.

Es bedarf einer besseren Information der Versicherten über die Möglichkeit bzw. den Anspruch, am gHKS teilzunehmen, und dessen schmerzfreien Ablauf. Die Verbesserung der Information über Berechtigung und Ablauf ist mit einfachen Mitteln zu erzielen und kann der Unwissenheit Abhilfe schaffen. Zwar zeigt sich ein sprunghafter Anstieg des Anteils der Informierten ab 35 Jahren, dennoch weiß nach über einer Dekade gHKS ein Drittel der Berechtigten nicht, dass sie einen Anspruch auf das gHKS haben. Von Bedeutung scheint demnach auch das Alter der Screeningberechtigten zu sein, denn die Teilnahmequote ist bei den jüngeren deutlich niedriger. Hier könnten eine generell größere Distanz zum Thema Hautkrebs und eine geringere Betroffenheit der Jüngeren von Bedeutung sein. So ist in der entsprechenden Frage der Anteil der wegen Hautkrebs Besorgten in der jüngsten Altersgruppe (< 35 Jahre) deutlich geringer. Dies könnte beim Einstiegsalter in die Früherkennung (35 Jahre) eine Rolle spielen. Objektive Gründe könnten das in der Tat deutlich geringere Risiko für Hautkrebs sein, subjektive das geringere Gesundheits- und Präventionsbewusstsein, das sich auch in anderen Erhebungen findet [17].

Bei den Teilnehmern über 65 Jahren scheint sich eine Art risikostratifizierter Synergieeffekt mit anderen Vorsorgeuntersuchungen zu zeigen, da diese über den Zugang zum gHKS deutlich besser informiert waren. Diese Tatsache offenbart, dass eine bedeutende, aber zu lösende Aufklärungslücke besteht. Auch könnte der Anteil derjenigen, die nicht am gHKS teilnehmen, da sie die Abläufe der Untersuchung nicht kennen, durch gute Aufklärungsarbeit reduziert werden.

Neben den Fachgesellschaften und Berufsverbänden engagieren sich in Deutschland auch private Stiftungen, Pharmaunternehmen sowie Kosmetikhersteller bei der Aufklärung zum Thema Hautkrebs. Als Beispiele sind „Euromelanoma“, geführt von einem Netzwerk europäischer Dermatologen in Unterstützung verschiedener Pharmafirmen, und die Aufklärungskampagne „Rebel against Skincancer“ von dem österreichischen Verein „Spot the Dot“, ebenfalls unterstützt von einer Pharmafirma, anzuführen [18, 19]. Hier wurde bereits erkannt, dass bestimmten Gruppen besondere Aufmerksamkeit geschenkt werden muss.

Eine zentrale Bedeutung für die Aufklärung zum Hautkrebs haben die Arbeitsgemeinschaft Dermatologische Prävention (ADP) e. V. und die Nationale Versorgungskonferenz Hautkrebs (NVKH) e. V.. Bereits seit 1989 engagiert sich die ADP in Zusammenarbeit mit der Stiftung Deutsche Krebshilfe bei der Aufklärung der Bevölkerung zur Prävention von Hautkrebs. Die NVKH verfolgt mit Medizinern und politischen Entscheidungsträgern ebenfalls mit ihren Projekten die Prävention und Versorgung von Hautkrebs.

Auch die jährliche Maßnahme „2 m^2^ Haut“ des Hauptverbandes der Berufsgenossenschaften in den deutschen Betrieben ist für die Prävention von hoher Bedeutung. Revolutionär engagierten sich bei dieser Kampagne erstmals Unfallversicherungsträger und die GKV in Kooperation. Der Erfolg der Kampagne verblasst allerdings mit den Jahren und bedarf einer neuen Auflage, um erneut in den Köpfen der Betroffenen präsent zu sein.

In der Befragung zeigte sich, dass das männliche Geschlecht und Menschen unter 65 Jahren eine höhere Wahrscheinlichkeit für die Nichtteilnahme haben. Dies sollte bei der Aufklärung besonders berücksichtigt werden. Im Rahmen der Aufklärungskampagnen zur „Männergesundheit“ der Bundeszentrale für gesundheitliche Aufklärung werden Männer zwar als „oft weniger gesundheitsbewusst als Frauen“ bezeichnet, explizit auf das Thema Haut, Hautgesundheit oder Hautkrebs wird allerdings nicht hingewiesen [20]. Dies deutet den Nachholbedarf seitens der Gesundheitspolitik an. Die GKV dient hier als Bindeglied mit direktem Zugang zum Patienten und damit als Informationsmedium. Es existieren zwar schon Informationsmöglichkeiten und -kampagnen über die einzelnen GKVen, nicht zu unterschätzende Kampagnen wie das Bonushefte-Programm für Vorsorgeuntersuchungen, das einige Patienten auch an das gHKS herangeführt hatte, wurden jedoch zum Großteil wieder eingestellt. Das in dieser Befragung aufgezeigte Informationsdefizit demonstriert, dass die Aufklärungsarbeit durch die GKV durchaus ausbaufähig ist und von den Krankenversicherern als Gesundheitsinformationsmedium offensiver angegangen werden sollte.

Mit der Aufnahme der Dermatoskopie in den EBM-Leistungskatalog der GKV zum April 2020 sollte ein Schritt in die richtige Richtung gemacht werden. Die sinnvolle Zusatzuntersuchung mit dem Dermatoskop war bisher als individuelle Gesundheitsleistung (IGeL) verzeichnet, und das gHKS wurde durch viele Ärzte nur in Kombination mit der IGeL angeboten. Die Aufnahme in den Leistungskatalog der GKV nimmt der Untersuchung also abschreckende Zusatzkosten für den Teilnehmer. Anzumerken ist, dass mit der neuen EBM-Ziffer kein Qualitätsnachweis der Dermatoskopie vom Untersuchenden und vom Untersuchungsgerät gefordert wird. Qualitätssichernde Maßnahmen sind hier also nachzufordern. Die Vergütung der aktualisierten EBM-Ziffer 01745 für Dermatologen wird von einigen Ärzten als unzureichend empfunden und deshalb scharf kritisiert, zum Teil auch boykottiert [21]. Damit wird das essenzielle Informationsmedium Arzt unfreiwillig beschnitten.

Insgesamt bedarf es besserer Informationen für die Versicherten über das gHKS und ihre Berechtigung dazu. Insbesondere die Gruppen männlich und mittleren Alters müssen besser erreicht werden. Aufklärungskampagnen müssen intensiviert werden, um Risikobewusstsein zu schaffen. Diese Aufgabe obliegt besonders der Gesundheitspolitik in produktiver Zusammenarbeit mit den Krankenversicherern und der Ärzteschaft. Die immer wieder beobachtete hohe Zustimmung der Gescreenten, die auch in der vorliegenden Studie gefunden wurde, ist dabei auch in der Öffentlichkeitsarbeit zu betonen. Zu vermitteln ist ferner die Einsicht, dass der Nutzen des gHKS nicht nur in der Verbesserung der Mortalität liegt, sondern gemäß dem Beschluss des Gemeinsamen Bundesausschuss auch in der Senkung der Morbidität, Erhöhung oder Wahrung der Lebensqualität und Minderung von Kosten durch Hautkrebs [4]. Nur in der ganzen Breite dieser Nutzenkriterien und in Gesamtsicht auf Melanom und hellen Hautkrebs sollte die Nutzendiskussion geführt werden.

## Fazit für die Praxis

- Die Besorgnis um das Thema Hautkrebs in der Bevölkerung sinkt.
- Seit der Einführung des gesetzlichen Hautkrebsscreenings hat etwa die Hälfte der berechtigten Befragten daran teilgenommen.
- Eine risikogruppenorientierte Aufklärung der Bevölkerung über die Relevanz des Themas Hautkrebs ist indiziert. Insbesondere die Gruppen männlich und mittleren Alters müssen besser erreicht werden.
- Der Nutzen des gesetzlichen Hautkrebsscreenings (gHKS) liegt nicht nur in der Reduktion der Mortalität, sondern auch in der Senkung der Morbidität, Erhöhung oder Wahrung der Lebensqualität und Minderung von Kosten durch Hautkrebs.
